# Umbilical Cord Mesenchymal Stem Cell-Derived Apoptotic Extracellular Vesicles Improve 5-FU-Induced Delayed Wound Healing by Mitochondrial Transfer

**DOI:** 10.3390/pharmaceutics17040453

**Published:** 2025-04-01

**Authors:** Hongbin Lai, Ling Lin, Yanrui Pan, Boqun Wang, Lan Ma, Wei Zhao

**Affiliations:** 1 Hospital of Stomatology, Guanghua School of Stomatology, Sun Yat-Sen University, Guangzhou 510055, China; laihb3@mail2.sysu.edu.cn (H.L.); linling23@mail2.sysu.edu.cn (L.L.); panyr5@mail2.sysu.edu.cn (Y.P.);; 2 South China Center of Craniofacial Stem Cell Research, Guangdong Provincial Key Laboratory of Stomatology, Sun Yat-Sen University, Guangzhou 510055, China

**Keywords:** delayed wound healing, 5-fluorouracil, umbilical cord mesenchymal stem cell-derived apoptotic vesicles, mitochondrial transfer, skin mesenchymal stem cells

## Abstract

**Background/Objectives:** This study aimed to explore the therapeutic potential of umbilical mesenchymal stem cell-derived apoptotic vesicles (UMSC-apoVs) in a 5-Fluorouracil (5-FU)-induced impairment in skin wound healing. **Methods:** UMSC-apoVs were isolated from UMSCs using differential centrifugation after the induction of apoptosis. A murine model was established by administering 5-FU via intravenous tail injection, followed by full-thickness skin wound creation. Mice received local injections of UMSC-apoVs at the lesion site. Wound healing was evaluated based on wound closure rates, histological analysis, and in vivo/in vitro functional assays. Rotenone (Rot)-pretreated UMSC-apoVs were used to explore the role of mitochondrial transfer between skin mesenchymal stem cells (SMSCs) and UMSC-apoVs in wound healing. **Results:** UMSC-apoVs significantly accelerated wound healing in 5-FU-treated mice, as demonstrated by enhanced wound closure rates and histological findings of reduced inflammatory infiltration and increased collagen deposition. UMSC-apoVs transferred mitochondria to SMSCs, enhancing viability, proliferation, and migration while reducing reactive oxygen species (ROS) production in SMSCs. Rot pretreatment inhibited the therapeutic effects of UMSC-apoVs on wound healing by inducing mitochondrial dysfunction in UMSC-apoVs. **Conclusions:** Our findings indicate that UMSC-apoVs improve 5-FU-induced impaired skin wound healing by facilitating mitochondrial transfer, suggesting a novel therapeutic strategy for alleviating chemotherapy-induced impairment in wound healing.

## 1. Introduction

Wound healing is a complex, multiphase process that restores the skin’s integrity and functionality. However, chemotherapy, a standard treatment modality for malignancies, often disrupts this process, leading to delayed wound closure [1,2,3]. Preoperative chemotherapy, particularly 5-fluorouracil (5-FU) treatment, can lead to delayed re-epithelialization, higher infection rates, and greater wound dehiscence [4,5]. As a thymidylate synthase inhibitor, 5-FU blocks DNA synthesis, arrests cells in the S-phase, and generates excessive reactive oxygen species (ROS), all of which contribute to delayed wound healing [3,6,7,8].

Traditional therapies, such as debridement and antimicrobial dressings, only alleviate superficial symptoms but do not effectively counteract the oxidative stress and dysregulated inflammation caused by chemotherapy [5,6,9,10]. Recently, mesenchymal stem cell (MSC)-based regenerative therapies have shown promise in tissue regeneration due to their immunomodulatory and regenerative properties [9,11,12]. Despite their advantages, the clinical application of MSCs remains limited by challenges such as poor cell survival in hostile microenvironments, poor engraftment, and potential immunologic rejection [13]. These limitations are particularly evident in chemotherapy-induced delayed wound healing, where systemic immunosuppression and extensive tissue damage exacerbate the already impaired regenerative capacity of MSCs. Therefore, there is an urgent need to develop innovative therapeutic approaches for chemotherapy-induced delayed wound healing.

Recently, extracellular vesicles (EVs) derived from MSCs have emerged as a novel cell-free therapeutic strategy. These vesicles preserve the regenerative potential of their parental cells, with a high physicochemical stability and biocompatibility [14,15]. Emerging evidence has indicated that EVs transfer mitochondria to host cells with mitochondrial dysfunctions, restoring the cell’s bioenergetics and suppressing oxidative stress levels [16,17,18]. Apoptotic vesicles (apoVs) represent a distinct subset of EVs generated during programmed cell death. Compared to EVs secreted by healthy cells, apoVs are enriched with a broader spectrum and higher abundance of bioactive cargo, including functional proteins and mitochondria [19,20,21,22]. Moreover, apoVs are easier to produce in large quantities, due to the controllable and efficient induction of apoptosis. They exhibit broader size distributions and unique molecular signatures. Notably, apoVs carry apoptosis-specific components such as phosphatidylserine (PS) and death-associated ligands. These molecules act as “eat me” signals to facilitate rapid recognition and engulfment by the recipient cells. These characteristics contribute to their superior regenerative, immunomodulatory, and anti-inflammatory effects in tissue repair [23,24,25]. Since 5-FU causes oxidative stress and mitochondrial dysfunction to impair wound healing [2], the delivery of mitochondria from apoVs may offer a promising solution [26,27]. Previous studies have identified umbilical mesenchymal stem cell-derived apoptotic vesicles (UMSC-apoVs) as promising therapeutic agents for chronic wounds such as diabetic wounds [14,19]. However, their efficacy in chemotherapy-induced skin damage remains unknown.

This study explores the therapeutic potential of UMSC-apoVs in a 5-FU-induced delayed wound healing model. Our findings demonstrate that UMSC-apoVs promote wound repair by restoring skin mesenchymal stem cell (SMSC) functions and redox balance through mitochondrial transfer. UMSC-apoVs may hold promise in mitigating chemotherapy-induced wound healing complications.

## 2. Materials and Methods

### 2.1. Mice

Male C57BL/6J mice (6–8 weeks, 18–22 g) were obtained from the Laboratory Animal Center of Sun Yat-sen University (Guangzhou, China). The mice were housed in a pathogen-free environment (24 °C, 12 h light/dark cycle, 50% humidity) with free access to food and drink. All procedures were approved by the Institutional Animal Care and Use Committee of Sun Yat-sen University (SYSU-IACUC-2024-002631).

### 2.2. Cell Culture and UMSC Characterization

#### 2.2.1. Isolation and Flow Cytometric Analysis of UMSCs

The UMSCs used in this study, as well as the related experiments, were approved by the Medical Ethics Committee of the Hospital of Stomatology, Sun Yat-sen University (KQEC-2021-59-01). As reported in our previous reports [20], UMSCs were isolated from full-term umbilical cords obtained through cesarean section, with informed consent from the donors. Cells were cultured in alpha Minimum essential medium (α-MEM, Biological Industries, Haifa, Israel) supplemented with 15% fetal bovine serum (FBS, Gibco, Thermo Fisher Scientific, Waltham, MA, USA), 2 mM L-glutamine, 100 U/mL penicillin, and 100 μg/mL streptomycin (all from Thermo Fisher Scientific, Waltham, MA, USA) at 37 °C with 5% CO_2_. Once colonies were established, the cells were trypsinized and passaged. Cells at passages 3 to 7 were used for experiments. Flow cytometric analysis (NovoCyte, Agilent Technologies, Santa Clara, CA, USA) was performed to assess the expression of mesenchymal markers CD90, CD44, and CD73, as well as hematopoietic markers CD45 and CD34 (1:100, all reagents from BioLegend, San Diego, CA, USA) [28].

#### 2.2.2. Multipotency Validation

The UMSCs’ multipotency was confirmed by osteogenic and adipogenic differentiation, as previously described [29]. Briefly, for osteogenesis, 1 × 10^5^ cells/well (6-well plates) were induced with osteogenic medium containing 100 μM L-ascorbic acid 2-phosphate (Sigma-Aldrich, St. Louis, MO, USA), 2 mM β-glycerophosphate (Sigma-Aldrich, St. Louis, MO, USA), and 10 nM dexamethasone (Sigma-Aldrich, St. Louis, MO, USA) for 14 days and then stained with 1% Alizarin Red S (Sigma-Aldrich, St. Louis, MO, USA). The Alizarin Red-positive areas were visualized using an inverted fluorescence microscope (Axio Observer.5, Zeiss, Oberkochen, Germany).

Amounts of 500 nM hydrocortisone (Sigma-Aldrich, St. Louis, MO, USA), 500 nM isobutylmethylxanthine (Sigma-Aldrich, St. Louis, MO, USA), 100 nM L-ascorbic acid phosphate, 10 μg/mL insulin (Sigma-Aldrich, St. Louis, MO, USA), and 60 μM indomethacin (Sigma-Aldrich) were used for adipogenic induction. After 28 days, the cells were stained with Oil Red O (Sigma-Aldrich, St. Louis, MO, USA). Images of positively stained cells were acquired by inverted fluorescence microscope.

Colony-forming units (CFUs) were assessed by seeding 2 × 10^2^ cells in 6-well plates for 14 days, fixed with 4% paraformaldehyde (Electron Microscopy Sciences, Hatfield, PA, USA) and stained with 0.5% crystal violet (Sigma-Aldrich, St. Louis, MO, USA) for visualization.

### 2.3. Isolation and Characterization of UMSC-apoVs

#### 2.3.1. Isolation

UMSCs at 100% confluence were incubated in α-MEM with 500 nM staurosporine (STS, ALX-380-014, Enzo Life Sciences, Farmingdale, NY, USA) without FBS for 6 h. The morphology of apoptotic cells at 0 and 6 h was captured using the oPERETTA CLS High-Content Imaging System (PerkinElmer, Waltham, MA, USA). ApoVs were isolated by centrifugation (800× *g*, 10 min, 4 °C; 2000× *g*, 10 min, 4 °C), filtered (5 μm), and pelleted at 16,000× *g* for 30 min at 4 °C [30,31].

#### 2.3.2. Transmission Electron Microscopy (TEM) and Nanoparticle Tracking Analysis (NTA)

For TEM, 10 µg/mL UMSC-apoVs was applied to copper grids, stained with 2% uranyl acetate (3 min), dried (30 min, room temperature), and imaged with a JEM-1200EX (JEOL, Tokyo, Japan). For NTA, UMSC-apoVs were suspended in filtered phosphate-buffered saline (PBS, BioWest, Nuaillé, France). The particle size distribution and potential were determined using the ZetaView PMX120 (Particle Metrix, Inning am Ammersee, Germany). Data analysis was performed with ZetaView software (version, 8.02.31).

#### 2.3.3. Nanoflow Cytometry (nFCM) and Immunofluorescent Staining

UMSC-apoVs were diluted in PBS and analyzed using nanoflow cytometry (NanoFCM, Xiamen, China) according to the manufacturer’s protocol. The samples were diluted, resulting in a particle count within the optimal range of 4000–10,000. To analyze the proportion of fluorescent intensity, the UMSC-apoVs were stained with Annexin V-FITC (1:100, Cell Signaling Technology, Danvers, MA, USA), calreticulin (1:100, Cell Signaling Technology, MA, USA), or cleaved caspase 3 antibodies (1:100, Cell Signaling Technology, Danvers, MA, USA), followed by Alexa Fluor 488-conjugated secondary antibody (1:200, Thermo Fisher Scientific, Waltham, MA, USA) staining for 1 h.

For immunofluorescence, the apoVs were stained as above and counterstained with CellMask (Invitrogen, Carlsbad, CA, USA) to visualize the vesicle membranes. The fluorescence images were acquired using a Zeiss Elyra 7 with Lattice SIM (Zeiss, Oberkochen, Germany), and the acquired data were analyzed using Zen 2.3 SP1 software (blue edition, Zeiss, Oberkochen, Germany).

### 2.4. Isolation and Multipotency Validation of SMSCs

SMSCs were isolated and cultured as described in previous reports [30,32]. Briefly, murine skin tissues were carefully minced and digested with collagenase type I (2 mg/mL; Worthington Biochemical, Lakewood, NJ, USA) and dispase II (4 mg/mL; Roche Diagnostics, Mannheim, Germany) for 1 h. The resulting single-cell suspensions were passed through a 70 μm strainer (BD Biosciences, Franklin Lakes, NJ, USA). All nucleated cells were cultured in α-MEM supplemented with 20% FBS, 2 mM L-glutamine, 55 μM 2-mercaptoethanol, 100 U/mL penicillin, and 100 μg/mL streptomycin (Invitrogen, Carlsbad, CA, USA). The self-renewal capacity of SMSCs was confirmed via CFU assays. Moreover, these SMSCs exhibited multipotent differentiation potential, as demonstrated by osteogenic and adipogenic differentiation assays.

### 2.5. UMSC-apoV Labeling and Internalization

SMSCs (5 × 10^4^ cells/well, 24-well plates) were incubated with PKH26-labeled UMSC-apoVs for 24 h (37 °C, 5% CO_2_). Then, cells were fixed (4% paraformaldehyde, 15 min), permeabilized (0.1% Triton X-100, 10 min), and stained with Alexa Fluor 488-phalloidin (Thermo Fisher Scientific, Waltham, MA, USA). Nuclei were counterstained with DAPI-containing mounting medium (Vector Laboratories, Burlingame, CA, USA). Fluorescence imaging was performed using a laser scanning confocal microscope (LSM 980, Zeiss, Oberkochen, Germany).

### 2.6. MitoTracker Staining of UMSC-apoVs

Mitochondria were stained with 100 nM MitoTracker Green (Beyotime, Shanghai, China), Hoechst (Beyotime, Shanghai, China), and CellMask. After staining, vesicles were centrifuged and resuspended in 20 μL PBS. The fluorescence images were acquired using a Zeiss Elyra 7 with Lattice SIM to visualize mitochondrial presence within the apoVs.

### 2.7. Co-Culture for UMSC-apoVs and SMSCs with Mitochondrial Staining

SMSCs were seeded in 6-well plates and cultured until reaching approximately 80% confluence. UMSC-apoVs were stained with MitoTracker Green (100 nM, 30 min) before being added to the SMSC culture. Following 24 h of co-culture, the SMSCs were collected, and the internalization of mitochondria-labeled UMSC-apoVs by SMSCs was quantitatively analyzed using flow cytometry.

To further confirm the internalization of mitochondria from UMSC-apoVs, SMSCs were seeded in confocal dishes (Nest, Wuxi, China), treated with 25 μM 5-FU for 12 h, and stained with MitoTracker Red (100 nM, 37 °C, 30 min) and Hoechst (10 min). UMSC-apoVs were pre-stained with MitoTracker Green (100 nM, 30 min) and co-cultured with SMSCs for 24 h, then imaged with a Zeiss Elyra 7 SIM.

### 2.8. Skin Wound Healing

C57BL/6 mice were randomly assigned to four experimental groups: PBS group, 5-FU group, 5-FU + UMSC-apoVs group, and 5-FU + rotenone (Rot)-pretreated-UMSC-apoVs group (*n* = 3). The 5-FU group, 5-FU + UMSC-apoVs group, and 5-FU + Rot-pretreated-UMSC-apoVs group received 15 mg/kg 5-FU (tail vein, days 1, 3, 5) [33]. On day 7, under anesthesia, a 1 cm × 1 cm dorsal wound was excised [29]. Treatments were injected locally at four wound corners with 5 × 10^6^ apoVs/100 μL PBS [20] or apoVs pretreated with 25 μM Rot (a mitochondrial complex I inhibitor, Macklin Chemical Technology, Shanghai, China) for 2 h. For Rot treatment, UMSC-apoVs were first resuspended in 100 μL of PBS, followed by the addition of an appropriate amount of Rot stock solution (10 mM in DMSO) to achieve a final concentration of 25 μM. The mixture was incubated at 37 °C for 2 h. Excess Rot was subsequently removed by washing with PBS and filtration, and the vesicle pellet was then resuspended in PBS for further use [34]. Wound closure was photographed (days 0, 3, 7, 10, 14) and analyzed using ImageJ software (version 1.53, NIH, Bethesda, MD, USA). The percentage of wound closure was calculated as the proportion of the remaining wound area relative to the initial wound size, providing a quantitative measure of healing dynamics.

### 2.9. Histological Staining

On postoperative day 14, full-thickness wound tissues with adjacent uninjured skin were harvested, fixed in 4% paraformaldehyde at 4 °C for 24 h following euthanasia, and processed for a graded ethanol dehydration series and paraffin embedding. Serial sections (5 μm thickness) were prepared for histological analysis. Standard hematoxylin and eosin (H&E) staining was performed to evaluate tissue recovery. Masson’s trichrome staining (Solarbio, Beijing, China) was utilized to visualize collagen deposition, following the manufacturer’s protocol [20]. To quantitatively evaluate the inflammatory response, immunohistochemical (IHC) staining was performed using primary antibodies against TNF-α (1:300, Abcam, Cambridge, UK), and IL-1β (1:300, Abcam, Cambridge, UK), followed by incubation with secondary antibody using the GTVision™ III Detection System/Mo&Rb (Including DAB) kit (Gene Tech, Shanghai, China). Images were analyzed quantitatively, and statistical comparisons were performed accordingly.

### 2.10. Cell Viability Assay

SMSCs (5 × 10^3^ cells/well, 96-well plates) were treated with 5-FU (0–25 μM; Sigma-Aldrich, St. Louis, MO, USA) for 24 h. SMSCs pretreated with 10 μM 5-FU for 24 h were then exposed to UMSC-apoVs (0–8 × 10^6^ particles/mL) for 24 h. Subsequently, the 5-FU-pretreated SMSCs were divided into three groups: (1) 5-FU alone, (2) 5-FU + UMSC-apoVs (4 × 10^6^ particles/mL), and (3) 5-FU + Rot-pretreated-UMSC-apoVs. Cell viability was assessed by adding 10 μL CCK-8 solution (Solarbio, Beijing, China) in 100 μL serum-free α-MEM, incubating them at 37 °C for 2.5 h, and measuring absorbance at 450 nm with a Synergy H1 microplate reader (BioTek, Winooski, VT, USA).

### 2.11. Ki67 Proliferation Assay

A cellular proliferation assay was conducted using a Ki67 staining kit (Beyotime, Shanghai, China) following the manufacturer’s protocol. SMSCs (5 × 10^4^ cells/well, 24-well plates) pretreated with 10 μM 5-FU (24 h) were co-cultured with 4 × 10^6^ apoVs or Rot-pretreated apoVs (2 h) for 24 h. Then, the cells were fixed, permeabilized, blocked (5% BSA, 30 min), and stained with anti-Ki67 primary antibody (1:200, Beyotime, Shanghai, China) overnight at 4 °C, followed by Alexa Fluor 488-conjugated secondary antibody (1:200). Fluorescence images were captured using an inverted fluorescence microscope and analyzed with ImageJ software.

### 2.12. Scratch Migration Assay

SMSCs (6-well plates) were pretreated with 10 μM 5-FU (24 h), scratched with a 200 μL pipette tip, and treated with UMSC-apoVs or Rot-pretreated UMSC-apoVs. Migration was imaged at 0, 12, and 24 h and analyzed using Image J software.

### 2.13. ROS Detection In Vitro and In Vivo

SMSCs (90% confluence, 6-well plates) were treated with 10 μM 5-FU (24 h), then co-cultured with 4 × 10^6^ UMSC-apoVs or Rot-pretreated UMSC-apoVs (24 h). ROS were detected with DCFH-DA (Solarbio, Beijing, China). Briefly, SMSCs were incubated with 10 μM DCFH-DA at 37 °C for 30 min in the dark, followed by Hoechst staining. After staining, the cells were washed three times with PBS to remove excess probe. Fluorescence images were acquired within 30 min to minimize the oxidation-related signal loss, using an inverted fluorescence microscope.

Skin tissue surrounding the wound was harvested. The harvested skin tissue was enzymatically digested to generate a single-cell suspension. The obtained cells were stained with 10 µM DCFH-DA at 37 °C for 30 min. Fluorescence intensity was assessed via flow cytometry.

### 2.14. Statistical Analyses

Statistical analyses were performed by GraphPad Prism 8 (GraphPad Software, Inc., San Diego, CA, USA). Comparisons between two groups were analyzed using independent unpaired two-tailed Student’s *t*-tests, and comparisons between more than two groups were analyzed using one-way ANOVA with the Bonferroni adjustment. All data are shown as the mean  ±  standard deviation (SD). *p*  <  0.05 was considered statistically significant.

## 3. Results

### 3.1. Isolation and Identification of UMSCs

**Figure 1:**
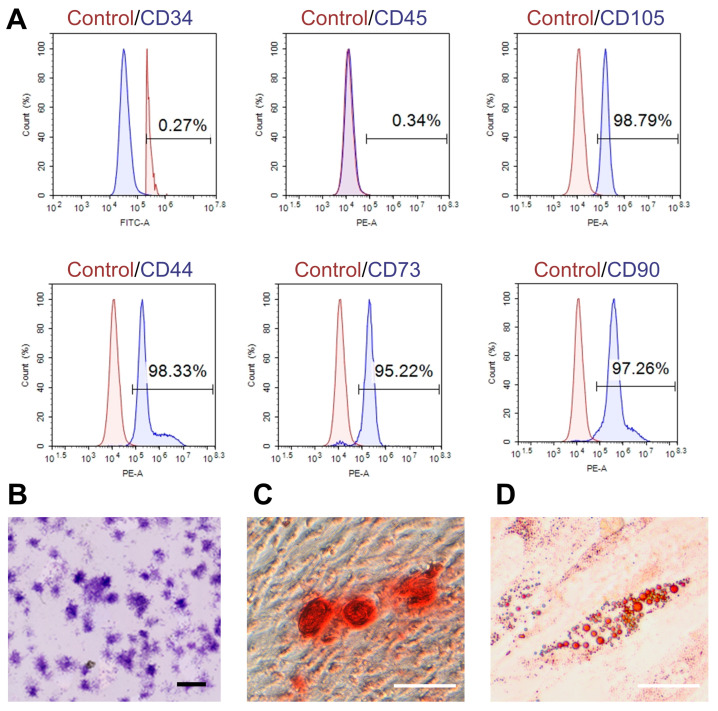
Characterization of UMSCs. (**A**) Flow cytometry analysis of UMSCs showing the expression of key surface markers, including CD34, CD45, CD44, CD73, CD90, and CD105; (**B**) CFU assay demonstrating the clonogenic potential of UMSCs (scale bar: 2000 μm); (**C**) Alizarin Red S staining indicating the osteogenic differentiation potential of UMSCs (red deposits indicate calcium accumulation, scale bar: 100 μm); (**D**) Oil Red O staining illustrating the adipogenic differentiation potential of UMSCs with lipid droplet accumulation (red staining marks intracellular lipid droplets, scale bar: 50 μm).

Flow cytometry analysis confirmed the MSC identity of UMSCs, demonstrating a high expression of MSC markers CD44, CD73, CD90, and CD105 (>95% positivity) and negligible expression of hematopoietic markers CD34 and CD45 (<5%) (Figure 1A). These results validated the phenotype of UMSCs with minimal hematopoietic contamination [19]. CFU assays demonstrated the strong clonogenic ability and self-renewal potential of UMSCs (Figure 1B). After osteogenic induction, significant mineralized nodule formation was observed in UMSC cultures according to Alizarin Red S staining (Figure 1C). Oil Red O staining showed a significant intracellular lipid droplet accumulation after adipogenic induction (Figure 1D). These findings revealed the multipotent differentiation potential of UMSCs.

### 3.2. Characterization of UMSC-apoVs

**Figure 2:**
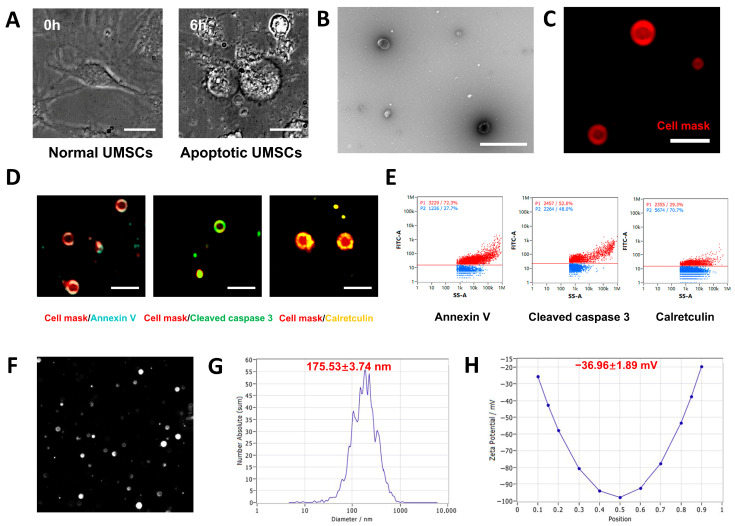
Characterization of UMSC-apoVs. (**A**) High-content imaging (40× magnification) showing the morphological changes in UMSCs at 0 and 6 h post-apoptosis induction; (**B**) TEM image of UMSC-apoVs (scale bar: 1 μm); (**C**) SIM image of PKH26-labeled apoVs (100× magnification; scale bar: 2 μm); (**D**) immunofluorescence staining of UMSC-apoVs markers (Annexin V, cleaved caspase 3, and calreticulin) (100× magnification; Scale bar: 2 μm); (**E**) nanoflow cytometry analysis showing the proportions of apoptotic markers (Annexin V, cleaved caspase 3, and calreticulin) on apoptotic vesicles. The red horizontal lines indicate gating thresholds used to distinguish marker-positive populations (P1, red) from marker-negative populations (P2, blue); (**F**) NTA image of apoptotic vesicles. The image was acquired using the ZetaView at a 10× objective magnification; (**G**) NTA analysis showing the mean size of UMSC-apoVs; (**H**) NTA analysis showing the mean zeta potential of apoptotic vesicles.

UMSC-apoVs were isolated from UMSCs following apoptosis induction with 500 nM STS for 6 h. STS-treated cells displayed apoptotic features, including cytoplasmic shrinkage, membrane blebbing, and detachment from the culture surface (Figure 2A). TEM and super-resolution structured illumination microscopy (SIM) imaging confirmed that the UMSC-apoVs displayed a typical double-membrane spherical structure (Figure 2B,C). SIM and nanoflow cytometry further revealed a high expression of apoptotic markers, which validated their apoptotic origin. SIM imaging demonstrated the presence of phosphatidylserine (PtdSer, detected by Annexin V binding), calreticulin, and cleaved caspase-3 (Figure 2D). Nanoflow cytometry further quantified these markers, demonstrating a 72.3% PtdSer positivity (shown by Annexin V binding), 29.3% calreticulin positivity, and 52% cleaved caspase-3 positivity (Figure 2E). NTA determined the median size of UMSC-apoVs to be 175.53 ± 3.74 nm, with a mean zeta potential of −36.96 ± 1.89 mV (Figure 2F–H). These findings confirmed that UMSC-apoVs were successfully isolated and identified.

UMSC-apoVs were isolated from UMSCs following apoptosis induction with 500 nM STS for 6 h. STS-treated cells displayed apoptotic features, including cytoplasmic shrinkage, membrane blebbing, and detachment from the culture surface (Figure 2A). TEM and super-resolution structured illumination microscopy (SIM) imaging confirmed that the UMSC-apoVs displayed a typical double-membrane spherical structure (Figure 2B,C). SIM and nanoflow cytometry further revealed a high expression of apoptotic markers, which validated their apoptotic origin. SIM imaging demonstrated the presence of phosphatidylserine (PtdSer, detected by Annexin V binding), calreticulin, and cleaved caspase-3 (Figure 2D). Nanoflow cytometry further quantified these markers, demonstrating a 72.3% PtdSer positivity (shown by Annexin V binding), 29.3% calreticulin positivity, and 52% cleaved caspase-3 positivity (Figure 2E). NTA determined the median size of UMSC-apoVs to be 175.53 ± 3.74 nm, with a mean zeta potential of −36.96 ± 1.89 mV (Figure 2F–H). These findings confirmed that UMSC-apoVs were successfully isolated and identified.

### 3.3. UMSC-apoVs Deliver Mitochondria to SMSCs

**Figure 3:**
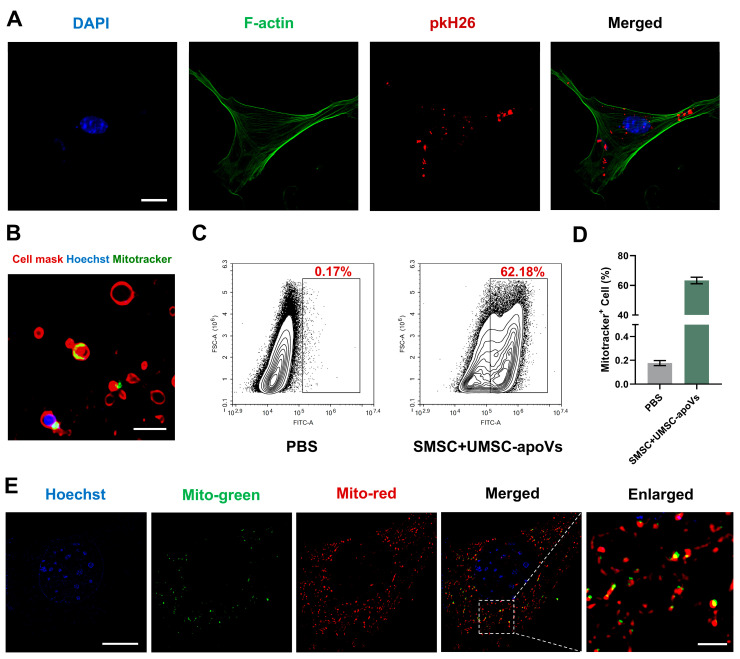
Mitochondrial transfer from UMSC-apoVs to SMSCs. (**A**) SIM image showing SMSCs internalizing UMSC-apoVs, labeled with PKH26 (100× magnification; scale bar: 20 μm); (**B**) immunofluorescent staining images showing mitochondrial staining in UMSC-apoVs (MitoTracker Green) (100× magnification; scale bar: 2 μm); (**C**,**D**) quantitative analysis by flow cytometry showing mitochondrial transfer from UMSC-apoVs to SMSCs; (**E**) co-culture of SMSCs with UMSC-apoVs, showing colocalization of mitochondria in both SMSCs and UMSC-apoVs as observed under Elyra 7 Lattice SIM (100× magnification; scale bar: 10 μm). Hoechst (blue) labels nuclei, MitoTracker Green (green) labels mitochondria in SMSCs, and MitoTracker Red (red) labels mitochondria in UMSC-apoVs. The dashed box in the merged image indicates the area shown at higher magnification (scale bar: 2 μm).

SMSCs were isolated and showed the capacity for self-renewal and multipotent differentiation (Supplementary Figure S1). After 24 h of co-culture, PKH26-labeled UMSC-apoVs were internalized by SMSCs (Figure 3A). We found the presence of mitochondria within UMSC-apoVs according to MitoTracker staining (Figure 3B). Flow cytometry analysis quantitatively confirmed mitochondrial transfer, showing a remarkable increase in MitoTracker Green-positive SMSCs after co-culture with UMSC-apoVs (62.18%), compared to the control group (0.17%) (Figure 3C,D). To further evaluate mitochondrial transfer from UMSC-apoVs to SMSCs, SMSCs were labeled with MitoTracker Red, and UMSC-apoVs were labeled with MitoTracker Green. SIM imaging revealed that mitochondria from UMSC-apoVs were colocalized with the mitochondria of SMSCs (Figure 3E). These findings suggest that mitochondria within UMSC-apoVs can be transferred to SMSCs.

### 3.4. UMSC-apoVs Improved 5-FU-Induced Delayed Wound Healing by Mitochondrial Transfer

**Figure 4:**
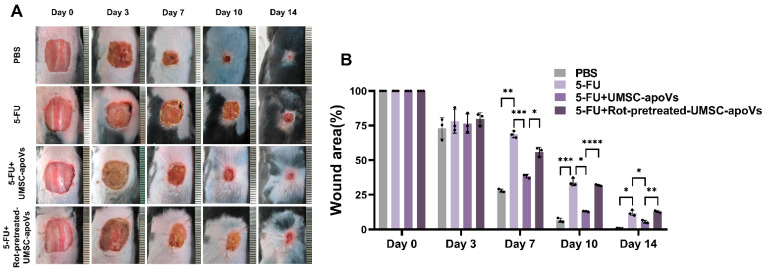
UMSC-apoVs transfer mitochondria to enhance wound healing of 5-FU skin wound model. (**A**) Representative images of dorsal wounds in different treatment groups on days 1, 3, 7, 10, and 14 post-wounding. (**B**) Quantification of wound area percentage at different time points. Data are presented as mean ± SEM (*n* = 3 per group). Statistical significance was determined by one-way ANOVA with Tukey’s post hoc test. * *p* < 0.05, ** *p* < 0.01, *** *p* < 0.001, **** *p* < 0.0001.

We then investigated the effects of UMSC-apoVs on 5-FU-induced delayed wound healing. Mice treated with 5-FU exhibited a significantly delayed wound closure compared to the PBS control group (Figure 4). The local administration of UMSC-apoVs markedly accelerated wound closure compared with the 5-FU-only group, with improved healing rates observed on days 7, 10, and 14 (*p* < 0.05). However, the pretreatment of UMSC-apoVs with Rot reversed their pro-healing effects.

**Figure 5:**
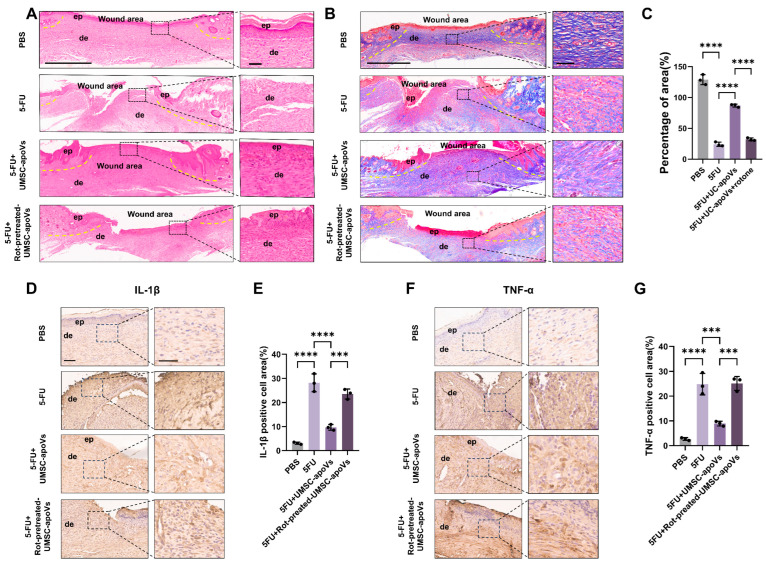
Histological analysis of 5-FU-induced delayed wound healing. (**A**) Hematoxylin and Eosin (H&E) staining of wound tissues on day 14, showing tissue architecture and inflammatory response across PBS, 5-FU, 5-FU + UMSC-apoVs, and 5-FU + Rot-pretreated-UMSC-apoVs groups. Yellow dashed lines delineate the wound margin. ep: epithelium, de: dermis (scale bar: 700 μm). Higher-magnification (scale bar: 50 μm). (**B**) Masson’s trichrome staining highlighting collagen deposition and dermal remodeling in the same groups. Yellow dashed lines indicate wound edges (scale bar: 700 μm). Higher-magnification insets (scale bar: 50 μm) illustrate cellular and extracellular matrix details. (**C**) Quantification of collagen deposition in the wound area based on Masson’s trichrome staining. (**D**) Immunohistochemical staining for IL-1β in wound tissues (scale bar: 200 μm). Higher-magnification insets (scale bar: 100 μm). (**E**) Quantification of IL-1β-positive staining area. (**F**) Immunohistochemical staining for TNF-α in wound tissues (scale bar: 200 μm). Higher-magnification insets (scale bar: 100 μm). (**G**) Quantification of TNF-α-positive staining area. Data are presented as mean ± SD (*n* = 3). *** *p* < 0.001, **** *p* < 0.0001, one-way ANOVA with Tukey’s post hoc test.

H&E and Masson’s trichrome staining results revealed distinct differences among the experimental groups. Compared to the PBS control group, mice treated with 5-FU displayed severe tissue disorganization, incomplete re-epithelialization, and a disrupted dermal structure, confirming the delayed wound healing induced by 5-FU. Local administration of UMSC-apoVs significantly enhanced wound repair relative to the 5-FU-only group, as demonstrated by a continuous, stratified epidermis and improved dermal architecture. However, in the 5-FU + Rot-pretreated-UMSC-apoVs group, wound healing was compromised, showing significantly diminished epidermal regeneration, and disorganized collagen deposition (Figure 5A–C). IHC staining showed that UMSC-apoVs decreased IL-1β and TNF-α levels in the wound tissues of 5-FU-treated mice. Rot pretreatment abolished these effects of UMSC-apoVs (Figure 5D–G). These findings confirmed that 5-FU impaired wound healing, while UMSC-apoVs promoted tissue regeneration by transferring functional mitochondria to SMSCs.

### 3.5. UMSC-apoVs Enhance Viability, Proliferation, and Migration of SMSCs by Mitchondrial Transfer

**Figure 6:**
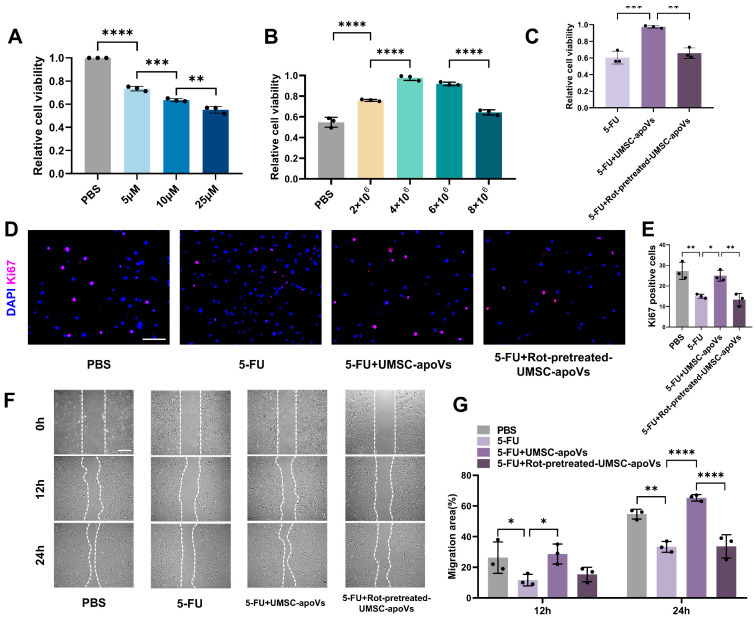
UMSC-apoVs promote the viability, proliferation, and migration of 5-FU-treated SMSCs by mitochondrial transfer. (**A**) CCK-8 assay showing SMSCs viability after treatment with 5-FU (0, 5, 10, 25 μM). (**B**) CCK-8 assay identifying the optimal UMSC-apoVs dose (0, 2 × 10^6^, 4 × 10^6^, 6 × 10^6^, 8 × 10^6^ particles/mL) after 24 h of co-incubation with 5-FU-pretreated SMSCs. (**C**) CCK-8 assay confirming mitochondrial involvement in UMSC-apoV-mediated protection against 5-FU toxicity, with Rot pretreatment (25 μM, 2 h). (**D**) Ki67 immunofluorescence staining of SMSCs across PBS, 5-FU, 5-FU + UMSC-apoVs, and 5-FU + Rot-pretreatment-UMSC-apoVs groups (Scale bar: 200 μm). (**E**) Quantification of Ki67-positive cells. (**F**) Scratch-wound assay showing SMSCs’ migration in different groups (scale bar: 400 μm). (**G**) Quantification of migration area at 12 and 24 h. Data are presented as mean ± SEM (*n* = 3 per group). Statistical significance was determined by one-way or two-way ANOVA with Tukey’s post hoc test. * *p* < 0.05, ** *p* < 0.01, *** *p* < 0.001, **** *p* < 0.0001.

We further explored the effects of UMSC-apoVs on the viability, proliferation and migration of SMSCs. CCK-8 assays showed that 5-FU treatment at concentrations of 5, 10, and 25 μM significantly reduced SMSCs viability. An amount of 10 μM 5-FU was selected for the subsequent experiments based on its intermediate effect (Figure 6A). An amount of 4 × 10^6^ UMSC-apoVs was identified as the optimal concentration for counteracting 5-FU-induced cytotoxicity (Figure 6B). The pretreatment of UMSC-apoVs with 25 μM Rot for 2 h can significantly diminish their cytoprotective effects (Figure 6C). Ki67 immunofluorescence staining indicated that 5-FU treatment significantly inhibited SMSC proliferation, which was markedly restored by UMSC-apoVs (Figure 6D,E). However, Rot pretreatment suppressed this proliferative recovery. As shown in Figure 6F,G, 5-FU significantly impaired cell migration, while UMSC-apoVs markedly enhanced migration over time. Rot pretreatment disminished this pro-migratory effect. These results highlighted the critical role of mitochondrial transfer in UMSC-apoV-mediated wound healing.

### 3.6. UMSC-apoVs Reduce 5-FU-Induced Oxidative Stress via Mitochondrial Transfer

**Figure 7:**
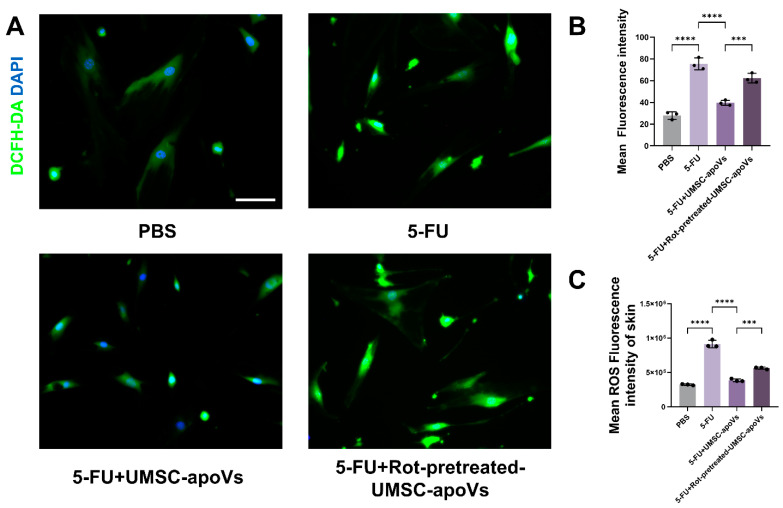
UMSC-apoVs reduce 5-FU-induced oxidative stress in SMSCs and wounded skin via mitochondrial transfer. (**A**) Representative immunofluorescence images of SMSCs stained with DCFH-DA, showing ROS levels across PBS, 5-FU, 5-FU + UMSC-apoVs, and 5-FU +Rot-pretreated-UMSC-apoVs (scale bar: 100 μm). (**B**) Quantification of intracellular ROS fluorescence intensity in SMSCs. (**C**) Flow cytometry analysis of ROS levels in skin cells from wounded tissues in the same groups. Data are presented as mean ± SEM (*n* = 3 per group). Statistical significance was determined by one-way ANOVA with Tukey’s post hoc test. *** *p* < 0.001, **** *p* < 0.0001.

Fluorescence microscopy revealed a significant increase in ROS levels in SMSCs treated with 5-FU, which was reduced by UMSC-apoV treatment (Figure 7A,B). Rot pretreatment can abolish this ROS reduction, confirming a mitochondrial-dependent mechanism (Figure 7A,B). Similarly, the flow cytometry analysis of skin cells from wounded tissues further confirmed that 5-FU significantly elevated intracellular ROS levels, while UMSC-apoV treatment reversed this elevation. Rot-pretreated UMSC-apoVs failed to mitigate intracellular ROS accumulation (Figure 7C). These results indicated that UMSC-apoVs alleviated 5-FU-induced oxidative stress and promoted wound repair by mitochondrial transfer.

## 4. Discussion

This study demonstrates the therapeutic potential of UMSC-apoVs in 5-FU-induced delayed wound healing. UMSC-apoVs transferred functional mitochondria to SMSCs, enhancing cellular viability, proliferation, and migration while reducing ROS levels, thereby accelerating the skin wound healing of 5-FU-treated mice.

In this study, we successfully established a murine model of 5-FU-induced delayed wound healing. Consistent with previous studies [2,4,8], our results confirmed that 5-FU impaired wound healing by reducing SMSCs’ viability, proliferation, and migration, while increasing ROS levels. In vivo, 5-FU delayed wound closure, exacerbated inflammation, and impaired re-epithelialization. Collectively, our findings confirm that chemotherapy impairs skin wound healing.

An excessive ROS accumulation and mitochondrial dysfunction are key contributors to 5-FU-induced wound healing impairment, so targeting oxidative stress and restoring mitochondrial function may offer an effective therapeutic approach [35,36]. While traditional interventions such as growth factors or MSC transplantation have shown some regenerative potential, their efficacy is often limited by harsh wound microenvironments and immunologic rejection risks [9,37]. Given these limitations, EVs, particularly apoVs, have emerged as promising cell-free therapeutic agents due to their ability to deliver bioactive molecules, including proteins, RNAs, and mitochondria, to recipient cells [19,21]. This delivery mechanism suggests that apoVs could counteract the adverse effects of 5-FU by reducing oxidative stress, strengthening cellular resilience, and promoting tissue regeneration.

ApoVs, a specific subset of EVs, hold great promise in promoting wound healing. Qu et al. reported that apoVs inherited SOX2 from pluripotent stem cells to accelerate wound healing [20]. Wang et al. demonstrated that UMSC-apoVs facilitated diabetic wound healing by inhibiting macrophage pyroptosis [19]. However, the effects of apoVs on chemotherapy-induced delayed wound healing remain unexplored. Our findings provide compelling evidence that UMSC-apoVs can effectively diminish 5-FU-induced viability, proliferation, and migration inhibition and reduced ROS levels in 5-FU-treated SMSCs. Histological analysis further demonstrated improved epithelial integrity, reduced inflammatory infiltration, and enhanced collagen deposition in UMSC-apoV-treated wounds, compared to the 5-FU-treated group. These results indicated that UMSC-apoVs may represent a novel and effective therapeutic strategy for chemotherapy-induced delayed wound healing.

A growing body of evidence has revealed the pivotal role of mitochondrial transfer in tissue regeneration [22,38]. It has been reported that EVs restore intracellular ATP production, alleviate oxidative stress, and enhance cellular metabolism by transporting functional mitochondria to host cells, thereby promoting tissue repair [38,39,40]. Our results showed that UMSC-apoVs contain mitochondria, as evidenced by SIM imaging. The uptake of these vesicles by SMSCs resulted in the colocalization of UMSC-apoV-derived mitochondria and SMSC-derived mitochondria, indicating successful mitochondrial transfer from UMSC-apoVs to SMSCs. As shown by DCFH-DA staining and flow cytometry, 5-FU elevated ROS levels in SMSCs. UMSC-apoVs significantly reduced the intracellular ROS accumulation, contributing to the improved viability, proliferation, and migration of SMSCs. Furthermore, Rot pretreatment, as a mitochondrial function inhibitor, abolished the therapeutic effects of UMSC-apoVs on wound healing according to in vivo and in vitro assays. These results demonstrated that UMSC-apoVs exerted their therapeutic effects through transferring functional mitochondria to SMSCs.

Despite these promising findings, certain limitations highlight avenues for future research. The specific molecular cargo within UMSC-apoVs responsible for mitochondrial transfer remains to be fully elucidated. The proteomic and transcriptomic profiling of UMSC-apoVs can be employed to explore their bioactive components. Moreover, the long-term fate of transferred mitochondria within recipient cells needs further investigation. Although the UMSC-apoVs used in this study were of human origin, they exhibited no observable immunogenicity in mice. This may be attributed to their apoptotic origin and phosphatidylserine exposure [23,24,25]. The safety and therapeutic efficacy of UMSC-apoVs should be further evaluated in human cells and larger animal models to facilitate clinical translation.

## 5. Conclusions

In summary, this study provides strong evidence that UMSC-apoVs improve 5-FU-induced delayed wound healing through promoting skin stem cell functions and mitigating oxidative stress by mitochondrial transfer. We propose UMSC-apoV treatment as a promising therapeutic approach for treating the delayed wound healing associated with chemotherapy.

## Data Availability

The original contributions presented in this study are included in the article/Supplementary Material. Further inquiries can be directed to the corresponding authors.

## Supplementary Materials

The following supporting information can be downloaded at: https://www.mdpi.com/article/10.3390/pharmaceutics17040453/s1, Figure S1: Characterization of SMSCs.
