# Supplementary material for: Umbilical Cord Mesenchymal Stem Cell-Derived Apoptotic Extracellular Vesicles Improve 5-FU-Induced Delayed Wound Healing by Mitochondrial Transfer
Source: Pharmaceutics. 2025 Apr 1;17(4):453. doi: 10.3390/pharmaceutics17040453 (PMC12030720; doi:10.3390/pharmaceutics17040453)
Supplement: Supplementary file 1 [file pharmaceutics-17-00453-s001.zip › pharmaceutics-3530973-supplementary.pdf]

Supporting information for

Umbilical Cord Mesenchymal Stem Cell-Derived Apoptotic Extracellular Vesicles Improve 5-FU-Induced Delayed Wound Healing by Mitochondrial Transfer

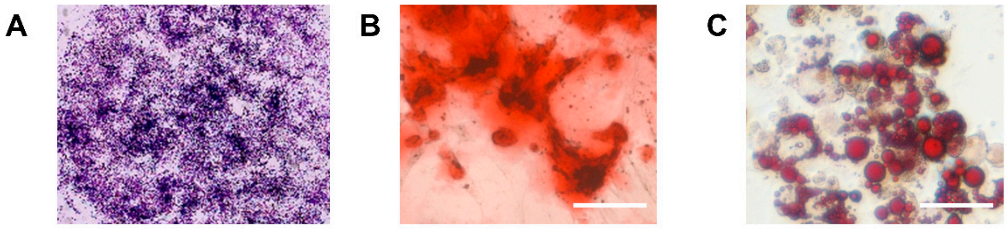

Figure Legends

Figure S1 Characterization of SMSCs. (A) CFU assay demonstrating the clonogenic potential of SMSCs; (C) Alizarin Red S staining indicating the osteogenic differentiation potential of SMSCs (Scale bar: 100 μm); (D) Oil Red O staining illustrating the adipogenic differentiation potential of SMSCs with lipid droplet accumulation (Scale bar: 50 μm).
